# Notes and News

**Published:** 1892-12-03

**Authors:** 


### NOTES AND NEWS.

COLLECTED AND COMMUNICATED.

A MEETING of the Surgical Aid Society will be held on December 9th, at three p.m., at Cannon Street Hotel, under the presidency of the Lord Mayor.

BETTER late than never. The authorities of the Nottingham General Hospital are evidently awakening to a realisation of their responsibilities, and efforts are being made to place the institution on a better footing. We predict that improvement in this respect will be followed by a like improvement in general interest and financial matters. We are glad to see that the remarks of our Commissioner on the occasion of his recent visit to Nottingham are receiving careful consideration, but unless his recommendation as to the entire reconstruction of the older and central portion of the hospital be carried out Nottingham cannot claim to possess an institution worthy of modern progress.

DR. BRIDGES, inspector of the Local Government Board, is about to retire from office. The announcement was made public at the last meeting of the Metropolitan Asylums Board, when Dr. Bridges made an excellent farewell speech. He pointed out that the Asylums Board had received severer criticism than they merited. Acquainted with the history of the development since 1868, he was in a position to make this statement. The original intention of the Board was to provide accommodation for the inmates of workhouses only. The vestries and local authorities of London soon learnt the superior accommodation the Board was providing on the occasion of a severe epidemic of small-pox, and threw on to their shoulders the care of patients who rightly had no claims upon them. Since that period the Board had been struggling to meet the ever-increasing demands made upon them, and Dr. Bridges stated that he was much struck by the readiness evinced to meet all requirements and adopt all improvements suggested by experience. Dr. Bridges' remarks were made in a spirit of fairness to the Board, and we hope that as he speaks with the authority of knowledge, the future efforts of this body may meet with encouragement as well as criticism. The provision for fever patients is attended with more difficulty than perhaps any branch of the public service, but this appears to be taken into too little account.

THE Hospital Saturday Fund now reaches the excellent total of £16,000, of which sum £11,000 has been contributed in weekly pence in the various industrial and business establishments of the metropolis.

AT the next meeting of The Hospitals Association, on December 7th, at eight o'clock, a paper will be read by C. Theodore Ewart, Esq., M.B., on "Epileptic Colonies." The meeting will take place in the board-room, Charing Cross Hospital. The subject is one commanding special attention at the present time, and an interesting discussion is expected.

IT is satisfactory to note that at the meeting of the Council of the Metropolitan Hospital Sunday Fund, on the 26th inst., it was unanimously resolved that on and from January 1st, 1893, the accounts of all institutions seeking a grant must be audited by a chartered accountant. It would be an immense gain to the voluntary hospitals if the public could be made to understand how remarkable is their efficiency, and that by the introduction of a uniform system of account- and of chartered accountants as auditors the business affairs of these institutions are now so conducted as to leave little, if anything, for reasonable people to carp at or criticise.

THE Homes for Little Boys (Farningham and Swanley) held their afternoon assembly at the Royal Albert Hall on Saturday, 26th ult., when there was a very large attendance. Mr. W. H. Willans, the treasurer, presided. Canon Barker and Dr. John Clifford were amongst the speakers. A large number of "school" and "workshop" prizes were awarded to the most deserving boys by Miss Willans. The boys looked remarkably well, bright and healthy and happy. When one thinks what they might have been save for these homes, one realises the value of the work done by the institution. There was an exhibition of the various trades taught at the homes, and also a display of musical drill. Canon Barker pleaded eloquently for the £2,000 required in order to avoid a curtailment of the society's operations. There are at present 500 boys in the homes, and they are all either orphans or homeless, and many are awaiting admission.

THE question of "To be or not to be" has just been settled at Chicago, in respect to the sale of intoxicating drinks at the World's Fair. During the discussion much diversity of opinion as to where the line should be drawn prevailed. The Commissioner for California adopted a highly moral tone of objection to all intoxicants, save the light wines of California. American criticism on the verdict is reassuring, if a little puzzling, to matter-of-fact English minds. We are reminded that although saloons and bars "are not" to be, and only "light and stimulating beverages" are to be sold in cafés and restaurants, that visitors of all nationalities will experience no difficulty in procuring "what they are accustomed" to. Thus it would appear that collectively the official mind of America entirely discourages that which it believes to be more harmful than beneficial. Experience, however, removes the dust from the eyes of the uninitiated, and we are thus forced to conclude that America is an example of the failure of legislation to solve the weighty drink question.

